# Mental Work Requires Physical Energy: Self-Control Is Neither Exception nor Exceptional

**DOI:** 10.3389/fpsyg.2018.01005

**Published:** 2018-07-05

**Authors:** Benjamin C. Ampel, Mark Muraven, Ewan C. McNay

**Affiliations:** ^1^Department of Psychology, University at Albany, State University of New York, Albany, NY, United States; ^2^Behavioral Neuroscience, University at Albany, State University of New York, Albany, NY, United States

**Keywords:** glucose, brain energetics, ego depletion, self-control, motivation, attention

## Abstract

The brain’s reliance on glucose as a primary fuel source is well established, but psychological models of cognitive processing that take energy supply into account remain uncommon. One exception is research on self-control depletion, where debate continues over a limited-resource model. This model argues that a transient reduction in self-control after the exertion of prior self-control is caused by the depletion of brain glucose, and that self-control processes are special, perhaps unique, in this regard. This model has been argued to be physiologically implausible in several recent reviews. This paper attempts to correct some inaccuracies that have occurred during debate over the physiological plausibility of this model. We contend that not only is such limitation of cognition by constraints on glucose supply plausible, it is well established in the neuroscience literature across several cognitive domains. Conversely, we argue that there is no evidence that self-control is special in regard to its metabolic cost. Mental processes require physical energy, and the body is limited in its ability to supply the brain with sufficient energy to fuel mental processes. This article reviews current findings in brain metabolism and seeks to resolve the current conflict in the field regarding the physiological plausibility of the self-control glucose-depletion hypothesis.

The human brain, though remarkable for its complexity, is still an energy-system subject to the laws of physics. Chief among these constraints is that no work can be performed without energy. Despite the seemingly self-evident nature of this premise, energy models of cognition have had a turbulent history since their conception. Partially product of the law of conservation of energy that was formulated in the 1840s, energy models of cognition initially took root in Freudian psychodynamics (e.g., Freud, 1960). However, they eventually faced strong resistance, being largely displaced by computational models that were product of the cognitive revolution (for a review, see Lykken, 2005). Despite the waning popularity of energy models of cognition, modeling of brain-energy supply was significantly improved in the 1970s and continues to be refined to this day (Lund-Andersen, 1979; Fox et al., 1988; Raichle and Mintun, 2006; Vaishnavi et al., 2010). In fact, it was the modeling of brain-energy supply that eventually resulted in modern fMRI and PET techniques (Sokoloff, 1977, 1979). Despite these advances, energetic accounts of cognition lay largely dormant in the social sciences until the late 1990s, when research on self-control depletion led to the proposals that: (1) the cognitive processes underlying the ability to inhibit, override, and alter our dominant responses are critically dependent on a physical, exhaustible energy reserve that is depleted by the prior exertion of self-control; and (2) self-control is different from other cognitive processes in this regard (Baumeister et al., 1998; Muraven and Baumeister, 2000).

Early research was generally supportive of the first of those proposals (i.e., that self-control cannot be maintained indefinitely, and performance suffers following prior exertion of self-control). Individuals who employ self-control do seem to exhibit marked reductions in self-control exertion during subsequent tasks (for overviews of research on the topic, see Hagger et al., 2010; Muraven, 2012). However, despite being initially well-replicated, the research on self-control depletion was surprisingly mute regarding potential mechanisms behind the depletion effect. In fact, the identity of the consumable, limited resource underlying self-control depletion was left largely ignored until the glucose model of self-control depletion (Gailliot and Baumeister, 2007).

While evidence for the glucose model was initially encouraging, several authors have since expressed doubts concerning its plausibility (e.g., Kurzban, 2010; Beedie and Lane, 2012; Kurzban et al., 2013). The arguments against this model of self-control have in some cases relied on erroneous accounts of brain glucose supply, usage, and the links between neural activity and glucose metabolism. Our primary goal in this paper is to correct these inaccuracies. In doing so, we give a brief primer on self-control depletion, and then describe claims made by the glucose model of self-control while addressing inaccuracies on both sides of the debate. Our goal is to place discussion of self-control depletion, and the impact of limited brain glucose supply on cognitive processing in general, on a sound biological foundation. We reach the general conclusion that a wide body of evidence from the field of brain metabolism and cognition supports the fact that glucose levels within specific brain areas can be acutely decreased by cognitive demand calling on those areas, and that such drainage places a limit on cognitive processing. However, we argue that there is nothing physiologically distinct about self-control and that – on the contrary – brain glucose depletion can be caused by a wide variety of cognitive tasks. Hence, we conclude that the glucose model of self-control depletion is flawed in asserting unique metabolic properties for the cognitive and neural processes mediating self-control, and that, in terms of glucose consumption, there is little or no support for drawing any distinction between the neural processes involved in self-control and those involved in other cognitive functions.

Importantly, despite being initially well replicated, there is currently some debate concerning the size of the depletion effect, with some scholars arguing that it is being overestimated (Carter and McCullough, 2014, p. 7), and others arguing that it is not (for a review on meta-analytic investigations of depletion, see Cunningham and Baumeister, 2016). Furthermore, it is possible that self-control depletion is not determined by inadequate brain glucose supply during exertion of self-control, and numerous theories have risen to explain the depletion effect *sans* brain glucose depletion (e.g., Inzlicht and Schmeichel, 2012; Kurzban et al., 2013; Ampel et al., 2016). Research has suggested that participants who gargle with glucose, but do not consume it exhibit attenuation of the depletion effect (e.g., Sanders et al., 2012; Carter and McCullough, 2013; Hagger and Chatzisarantis, 2013; but see Lange and Eggert, 2014; Boyle et al., 2016), and new meta-analyses go so far as to provide evidence that glucose administration does not actually ameliorate the depletion effect at all (Dang, 2016). These findings do not factor into our paper, as our objective is entirely concerned with addressing the inaccuracies that have occurred in the process of criticizing the biological plausibility of the glucose model of self-control depletion. We do not aim to review this recent literature, nor to resolve whether self-control depletion is, in fact, driven by reduced brain glucose levels: rather, we limit our discussion to evaluating (1) whether depletion of brain glucose can act to limit neural and cognitive processes, and (2) whether there is any support for processes underlying self-control being unique in this regard.

## Self-Control Depletion

Initial research on self-control depletion was largely atheoretical regarding a specific mechanism. Research found no evidence that the decline in self-control performance after exerting self-control was a product of mood, arousal, frustration, self-efficacy, or other psychological processes. Indeed, the only factor originally identified as related to the decline in self-control performance was the amount of self-control exerted initially (for a review, see Hagger et al., 2010; Muraven, 2012). Given these results, researchers (e.g., Muraven and Baumeister, 2000) suggested that exerting self-control might deplete a conceptual resource that they referred to as ego strength (alternatively called self-control strength). Under this model, the resource is limited, gets depleted in the processes of exerting self-control, and is only slowly replenished. Most critically, the level of this resource was suggested to be related exclusively to self-control outcomes: a reduction in this ‘resource’ causes poorer performance but only on tasks that require self-control.

Ongoing research has been focused on understanding the nature of this depletion process. Most notably, researchers have been trying to determine what the limited resource is. Some have suggested that much of the depletion process is driven by expectations – that is, people believe that self-control is bounded by a limited resource and thus act accordingly (Martijn et al., 2002; Job et al., 2010). On the other hand, other researchers have suggested a physiological basis for this resource. The most prominent of these resource models is the glucose model of self-control depletion (Gailliot and Baumeister, 2007), referred to here as ‘the glucose model.’

## The Glucose Model of Self-Control Depletion

The glucose model is comprised of two claims, which together propose brain glucose to be the physical manifestation of ego strength (Gailliot and Baumeister, 2007). The first is that glucose (as the primary energy source of the brain) is, under some conditions, used in some brain regions faster than it can be replenished, and that this replenishment delay creates limits on cognitive and neural function.

Importantly, seminal research on the glucose model, while initially promising, has since been the subject of some scrutiny. For example, studies have found that the mere taste of glucose can lead to attenuation of the depletion effect, even without consumption (e.g., Sanders et al., 2012; Carter and McCullough, 2013; Hagger and Chatzisarantis, 2013). Additionally, an analysis of the original work examining the glucose hypothesis (Gailliot et al., 2007) suggests that, given the power and effect size reported across all nine independent studies in the original paper, nine confirmations is more than is statistically plausible, indicating the possibility of selective reporting (Schimmack, 2012). Meta-analyses have added confirmatory evidence of a file-drawer problem in tests of the glucose model in general. Vadillo et al. (2016) performed a p-curve analysis on 19 tests of the glucose model and found that the number of significant confirmations of the glucose hypothesis was ill distributed given the reported size of the effect, suggesting that some, possibly many, may be false positives or the result of selective reporting. The potential for a file-drawer problem has found confirmatory evidence in other meta-analyses (Dang, 2016). These findings cast doubt on the validity of previous research in support of the glucose model of self-control depletion. However, we contend that the validity of previous research aside, arguing that the human brain is not highly sensitive to glucose depletion would be an overreaction, and one that would be overlooking over a century of research on brain glucose metabolism. In short, while the direct cause of self-control depletion may not be transient decreases in brain glucose, it is incomprehensible that there would be no relationship between brain glucose and self-control.

The second, more dramatic claim of the glucose model is that self-control processes are special regarding such glucose depletion. That is, glucose depletion is especially pronounced and impactful in areas responsible for self-control, as it is a particularly metabolically expensive process. Accordingly, the glucose model proposes that self-control is uniquely constrained by provision of glucose to the brain. We will examine these two distinct claims separately, starting with a brief summation of research on the relationship between brain glucose supply and neural activity, followed by an analysis of the most common critiques of brain glucose as limiting for cognitive performance, and finally, ending with an exploration of the second claim of the glucose model of self-control depletion.

### Brain Metabolism and Neural Activity

The glucose model of self-control depletion proposes that self-control depletion is caused by diminished glucose in the brain as a product of increased neural activity. This is a defensible hypothesis. There is a large body of evidence to support the claim that brain glucose supply can be diminished, and, in the process, can limit neural activity (resulting in decreases in cognitive ability).

The human brain is a physical system; like all physical systems, it requires energy to perform work. Furthermore, the human brain is much more energetically demanding than other organs. On a second by second basis, the human brain uses more energy at rest then a human thigh during a marathon (Hochachka, 1994). While only accounting for 2% of total body mass, the adult human brain uses roughly 20% of daily caloric intake. The brain is also highly selective in terms of fuel. Under normal, healthy physiological conditions, cognitive and neural processes are fueled solely by glucose. This glucose must be constantly supplied from the blood.

Early models of brain glucose supply initially assumed that the supply of glucose to the brain was always in excess of demand (Sokoloff, 1977, 1979, 1984; Lund-Andersen, 1979), but this posed a problem for attempts to explain the now well-replicated finding that administration of glucose can enhance cognitive processes (e.g., Gold et al., 1986, 1995; McNay et al., 2000; McNay and Gold, 2002). Indeed, direct measurements of *in vivo* brain glucose supply and metabolism during cognitive testing subsequently found that difficult cognitive tasks resulted in an increase in glucose metabolism that correlated with the difficulty of the task. This demand for glucose locally exceeded supply within many specific brain areas during and immediately following tasks that are mediated by those specific brain regions, leading to depletion of local glucose availability (McNay et al., 2000, 2001; De Bundel et al., 2009; Rex et al., 2009; Newman et al., 2011; Sandusky et al., 2013). Furthermore, the brain responds to increased neural activity by increasing local blood flow in order to replenish glucose: this is responsible for the transient increase in blood oxygenation that is the basis for fMRI spatial mapping of neural activity (Sokoloff, 1979).

Only one study (McNay et al., 2000) appears to have deliberately varied task difficulty while measuring local brain glucose levels. In that task, young rats were placed into a spontaneous alternation task (known to be hippocampally mediated) in one of two mazes: either 3- or 4-arm, with the latter causing a 50% greater load on spatial working memory. Performance on the 3-arm maze was at ceiling, unable to be enhanced by glucose, and caused only an 11% drop in hippocampal extracellular glucose; rats placed in the 4-arm maze had a drop in hippocampal glucose greater than 30%, which was reversed by a systemic dose of glucose that enhanced performance on the task. Measurements outside the hippocampus both in that study and a second (McNay et al., 2001) confirmed that the glucose depletion was localized to the hippocampus, rather than occurring brain-wide or even in adjacent brain regions. A second study also supports a correlation between task difficulty and the magnitude of brain glucose depletion: when aged rats were placed into the same 4-arm task, they showed markedly impaired performance compared to young rats, and a much greater depletion of hippocampal glucose (McNay and Gold, 2001); when the aged animals were treated with glucose, both the cognitive impairment and the hippocampal glucose depletion were reversed in a correlated manner.

Note that neither in these studies nor elsewhere have data been obtained about performance on subsequent tasks, whether self-control or otherwise. These are the data that would be needed to address the controversial issue of persistent self-control depletion. It is worth noting that the data from McNay et al. (2001) indicated a return to baseline hippocampal glucose levels in young animals within 5–10 min after completion of the maze task (likely in part reflecting a rise in glucose caused by increased arousal). This suggests that any such cognitive impairment would be relatively transient. However, this is speculation.

One body of evidence pertinent to the relationship between brain glucose supply and neural activity stems from research on acute hypoglycemia, a state characterized by a sudden and transient level of low blood glucose. Research investigating which cognitive functions are impaired when individuals are known to have low available glucose allows some induction concerning the brain’s natural ability to cope with the cost of increased mental activity. For example, driving is a task that requires complex motor control, as well as spatial processing and judgment. Furthermore, evidence suggests it places significant metabolic demand on requisite brain areas (e.g., Cox et al., 2002). Individuals who suffer from type 2 diabetes mellitus (and thus have a history of hypoglycemia) exhibit less motor control while driving, as evidenced by a higher collision rate and worse performance on driving simulators (Cox et al., 2003). Further evidence suggests that for individuals suffering acute hypoglycemia driving results in a significant increase in overall brain glucose consumption (17%; Cox et al., 2010).

Another method for studying cognition function during acute hypoglycemia is to induce it through the administration of insulin (i.e., a hyperinsulemic clamp). When normal healthy individuals are given insulin, it causes rapid absorption of glucose from the blood stream. This results in rapidly declining blood glucose levels leading to inadequate supply of glucose to the brain. Typically, participants exposed to a hyperinsulemic clamp undergo cognitive tests in conditions of acute hypoglycemia and then under conditions of euglycemia (i.e., normal glucose levels). Through this process, cognition under conditions of inadequate brain glucose can be studied. Utilizing this method, Anderson et al. (2006) induced normal healthy individuals into a hypoglycemic state, and then presented them with bright moving visual stimuli while measuring their brain response via fMRI. Several days later, they repeated the experiment with the exception that participants’ blood glucose was now held in a euglycemic state. When in a hypoglycemic state, participants exhibited less indication of energy metabolism in the visual cortex then when they were in a euglycemic state. This suggests that when the body is unable to adequately deliver glucose to the brain, reduced neural activity results.

In short, there is nothing empirically precarious in the proposal that cognitive processes are limited by restraints in brain glucose supply; brain extracellular glucose does decrease with cognitive activity, and the magnitude of depletion is correlated with activation of specific brain regions and cognitive processes. Moreover, systemic hypoglycemia does cause cognitive impairment despite blood glucose remaining well above normal brain-extracellular-fluid glucose levels, both in animal studies and in a large number of human studies across multiple cognitive domains (e.g., Cox et al., 1993; Weber et al., 1994; Draelos et al., 1995; Clarke et al., 1999; Strachan et al., 2000; McNay et al., 2010). Several papers in the field make the unfortunate error of confounding measurements of blood glucose with those of brain glucose (e.g., Gailliot and Baumeister, 2007; Gailliot et al., 2007, 2010; Kurzban, 2010; Beedie and Lane, 2012). We discuss this in some detail below, but it is important to note in particular that although systemic hypoglycemia causes neural glycopenia (i.e., insufficient brain glucose availability–because glucose supply from the blood is impaired), lack of fluctuation in blood glucose says nothing with regard to brain glucose depletion caused by cognitive demand (because brain glucose depletion does not affect, and is not reflected in, systemic glucose levels).

## Previous Critiques of Brain Glucose Limiting Cognitive Performance

The proposal that self-control depletion is a product of diminished glucose in the brain, and specifically, in brain region(s) mediating self-control processes (see Gailliot and Baumeister, 2007) has attracted criticism within the psychology literature. Interestingly, such criticism has focused on glucose depletion as a limitation on cognitive performance rather than on the proposed special nature of self-control, with opponents often arguing that the model is physiologically implausible (e.g., Kurzban, 2010; Beedie and Lane, 2012; Kurzban et al., 2013). Understandably, given the specialized nature of the literature, these critiques have sometimes lacked a comprehensive understanding of current research in brain glucose metabolism and supply. Similarly, both the original paper on the glucose model (Gailliot and Baumeister, 2007) and follow-up papers (Gailliot et al., 2007, 2009, 2010) may not be appropriately constrained by the large body of work on brain glucose metabolism, or that on the modulation of cognitive function by glucose supply (specifically, in the contention that self-control is a cognitive process distinct in the magnitude of its cost from other cognitive processes). This has contributed to a lack of clarity in this area, which we hope to rectify in the following section. In doing so, we examine some of the more popular criticisms of the physiological plausibility of the glucose model and explore them in light of the body of research that has been conducted in the somewhat removed field of brain glucose metabolism.

## Does Brain Energy Expenditure Decline as a Function of Cognitive Task

One of the more common arguments against the glucose model is that the human brain does not exhibit large enough increases in energy consumption to result in brain glucose deficits (e.g., Kurzban, 2010, p. 247; Beedie and Lane, 2012, p. 148). To illustrate, critics of the glucose model have asserted that the brain’s energy expenditure exhibits little change from rest, concluding that because visual processing is a metabolically expensive process, if glucose supply was a constraint on brain function then “seeing would feel effortful” (Kurzban et al., 2013, p. 647). Sidestepping the unspoken assumption that phenomenological sensation is indicative of cortical metabolic activity, visual processing was the basis for some of the earliest measurements of task-associated increases in local brain metabolism, and a substantial body of evidence has accumulated that, while seeing may not ‘feel’ effortful, high levels of optical stimulation do lead to localized reductions in brain glucose in the visual cortex (e.g., Wagner et al., 1981; Cooper, 2002; Béland-Millar and Messier, 2018). The idea that increases in cognitive functioning do not cause substantial localized increases in the brain’s requirements for glucose would invalidate fMRI measurements, major *in vivo* imaging techniques, and centuries of work on brain fuel supply. It would also suggest that some alternative explanation is needed for cognitive impairment during, for example, hypoglycemia (e.g., Cox et al., 1993; McAulay et al., 2001; Anderson et al., 2006).

## Does Brain Glucose Decline Enough to Cause Cognitive Impairment?

A second argument raised against glucose availability as a constraint on cognitive performance is that even if brain glucose levels were to decrease, this would nonetheless still leave sufficient glucose such that there would be little decline in cognitive or neural function. For example, Kurzban (2010) argues that the amount of glucose remaining in the blood after a self-control task is more than sufficient for the energy demands of additional exertion of self-control. Kurzban (2010) further reasons that the small amount of energy that the brain requires for self-control tasks is lower than the ∼3 calories provided by the artificially sweetened drinks commonly used as a placebo in studies testing self-control depletion (pp. 247–248) and thus, that there should be no functional difference in energy supply to the brain in studies where self-control is impaired versus where it is intact.

One contributor to confusion on this point may be that the majority of studies examining human brain metabolism and specifically self-control have relied upon measures of blood glucose as a proxy, and even go as far as to use the term blood glucose as if it were synonymous with brain glucose (e.g., Gailliot and Baumeister, 2007; Molden et al., 2012). It has been well-established for 40 years that glucose in the brain and blood forms distinct pools (e.g., Sokoloff, 1977, 1979, 1984; Lund-Andersen, 1979; Fellows et al., 1992), and this is again supported by direct measurement in animal studies. For instance, under conditions (a spatial working-memory task) that drain glucose in the hippocampus, blood glucose does not decrease (McNay et al., 2000, 2001) and in fact slowly increases in response to stress hormone release. Indeed, dissociation between brain and blood glucose levels is a consistent finding across studies that measure the two simultaneously (McNay et al., 2000, 2001; McNay and Sherwin, 2004b; De Bundel et al., 2009; Rex et al., 2009). In short, systemic glycemia is largely irrelevant to the likelihood of localized, transient brain glucose depletion, and it is this transient brain glucose depletion which causes deficits in cognitive processing. Some authors (e.g., Kurzban, 2010) have criticized the glucose model on the basis that self-control may not cause decreases in blood glucose. This is a misunderstanding: fluctuations in brain glucose, particularly when localized to specific brain regions involved in processing a cognitive task, do not affect blood glucose levels (see McNay et al., 2001). Conversely, alterations in systemic glucose (such as hypoglycemia, or delivery of a glucose drink) are reflected in brain glucose supply: the direction of causality is one-way.

Lastly, it may be helpful to address the specific “less than 1 calorie” estimated by Kurzban (2010) as a likely amount of energy being depleted by a self-control task, and the accompanying suggestion that this is too small an amount to be plausible as affecting cognitive performance. There are several errors involved in this conclusion, which are illustrative of the need for increased biological awareness in this sub-field of psychology. First, the calculation used may well err on the low side – for example, it assumes a maximal change in glucose consumption of 10%, whereas direct measurements show a reduction of local brain glucose that can be at least as high as 50% on even a relatively simple task (McNay et al., 2001). Second, to supply say 1 calorie to a specific brain region during a self-control task would require a much larger dose to be delivered to the individual: the amount arriving at a specific brain region during task performance would be limited by factors including (but not limited to) speed of metabolism, the fraction of systemic glucose delivered to the brain (generally taken as one-fifth), the specific brain region(s) involved as a fraction of the whole brain (unclear, but perhaps a further factor of one-tenth, generously; as Kurzban (2010) correctly notes, most cognitive tasks do not markedly affect total brain metabolism more than 1–10%), the efficacy of glucose transport from blood to neurons, and so on. Unless using direct intracranial microinjection, it is not possible to target the energy content of, for example, a sugary drink only to one brain region or system; rather, an amount several orders of magnitude larger is needed. Third, calories from a non-glucose source such as Splenda (Kurzban’s example) are irrelevant to the brain, which, under normal circumstances, uses only glucose as an energy source: non-glucose sugars are not transported into the brain, and hence cannot fuel the metabolic demands of cognition. So, for instance, delivering the commonly used dose of 250 mg/kg glucose found to enhance cognition in many of the human and animal studies cited above to a 60 kg individual requires 15 g of glucose, which provides 51 calories on a whole-body basis: the ballpark discussion above shows that on the order of 1–5 of those calories would arrive at a specific brain location. In other words, when placed in the correct whole-body context, even the numbers used by critics of the glucose hypothesis are consistent with the plausibility of glucose administration meeting the needs of cognitive demand. Fourth, note that in rat studies, when injected directly into brain regions processing a given task, doses of glucose several orders of magnitude lower than this have positive effects on cognitive processing (McNay and Gold, 1998; McNay et al., 2006a). Finally, and most importantly to the Splenda argument, the relevant variable is not any absolute amount of generic fuel: rather, it is whether cognitive tasks cause demand for glucose that exceeds supply; and then whether provision of exogenous glucose (specifically glucose) attenuates this supply-demand imbalance. Both of these are unequivocally established to be true, as we show throughout this paper, and are reflected in, for example, levels of neurotransmitter release specifically within the brain regions processing the task being performed (Ragozzino et al., 1998) in a manner that correlates with task performance.

In short, the brain is exquisitely sensitive to metabolic supply, and experimenters’ intuitions about the magnitude of changes in brain glucose supply and demand should be carefully grounded in available data regarding cognitive metabolism. One further example may serve to illustrate the need for correct biological framing in debate over the glucose hypothesis. Kurzban (2010) suggests that exercise (which consumes fuel) should hence diminish self-control if self-control requires brain glucose. On the contrary, exercise will increase blood flow (and hence brain glucose supply), elevate circulating epinephrine (well-established to enhance cognitive processes in a manner very similar to glucose (Hall and Gold, 1986; Morris et al., 2010; Morris and Gold, 2013), and have a host of other effects of which many enhance cognition [presumably including self-control – which is exactly what is found, as Kurzban (2010) notes], but is very unlikely to cause any decrease in brain glucose.

## Is Brain Glucose Exhaustion Evolutionarily Improbable?

A final critique of brain glucose depletion takes an evolutionary approach, arguing that brain glucose depletion would not be adaptive, and thus is unlikely to have been selected for in our ancestral past (e.g., Beedie and Lane, 2012). It is plausible, at least initially, that having sufficient glucose supply to the brain to support optimal neural functioning under normal circumstances might be adaptive, but there are two central problems with this argument. The first is that it underestimates the limits of evolution. While an interesting idea that certainly bears further investigation, the idea that it would be maladaptive for one intensive cognitive task to deplete glucose reserves mistakenly overlooks important facets of evolutionary processes. Darwinian notions of ‘unity of type’ and ‘developmental constraint’ bear relevance here. It is well-known that Darwin (1909) argued that we have a common ancestor, and that homologous anatomical/physiological properties (i.e., unity of type) descended from this common ancestor. Less well-known is the evolutionary notion of ‘developmental constraint,’ in which homologous structures acquired from distant ancestors act to constrain new adaptations. In other words, selection can only act on existing structures, and therefore each adaptation narrows and channels possible new variations. Thus, evolutionary theory posits that properties of an organism can be either a current adaptation to an environment or constraints imposed by past adaptations (Gould, 2002). In short, regardless of how advantageous it might be for elephants to evolve adaptations for jumping–they never will in their current state, as their developmental trajectory (i.e., size) restricts any possible variance in jumping ability. This certainly holds for humans as well. While perhaps, as Beedie and Lane (2012) propose, it may be uneconomic for intensive brain utilization to result in deficits in the ability to use that neural system, this does not mean it cannot be true. It only means that, if not an adaptation to the environment, it is likely a constraint posed by past adaptation. While Beedie and Lane (2012) are correct in their assertion that “… an adaptive psychological characteristic is more likely than a maladaptive one to propagate through the species” (p. 145), this does not imply that it is the only reason it may propagate throughout a species. As demonstrated, another reason is simply through limitations posed by past adaptations.

There are many possible reasons behind the human brain’s obligate reliance on glucose for energy. For instance, fuel has been scarce for most of evolutionary history: why has the brain evolved to be able to use only glucose? The context of scarcity may perhaps provide one evolutionary explanation for the limits on brain glucose supply: a need to avoid over-expenditure on brain metabolism (already very high compared to other organs). It is plausible, at least initially, that having sufficient glucose supply to the brain to support optimal neural functioning under normal circumstances might be adaptive; we discuss this below. However, extending this to a more general objection to neuronal firing being limited by glucose availability is implausible.

The second problem with this argument is that it oversimplifies the complex nature of brain-energy metabolism. Although it is ostensibly true that having glucose supply always exceed demand would be advantageous if there were no negative tradeoffs, this critique misses the well-established and marked neurological damage caused by chronic hyperglycemia, including neuronal over-excitation by elevated glucose levels (which can cause neuronal death, a process known as excitotoxicity), vascular damage, and oxidative stress. Moreover, chronic excess of glucose in the bloodstream over that required for baseline metabolism, including brain metabolism, is the defining symptom of type 2 diabetes: such hyperglycemia directly causes neuropathy and is directly linked not only to cognitive impairment but to development of dementia including Alzheimer’s disease (Craft et al., 1993; Draelos et al., 1995; Messier and Gagnon, 1996; Arvanitakis et al., 2004; Vincent et al., 2005; Li and Holscher, 2007; Moreira et al., 2007; Zhao and Townsend, 2009). Further, glucose regulation is part of a complex neuroendocrine system that involves many pro-cognitive molecules that would also be dysregulated by chronically increased glucose supply.

Beedie and Lane (2012) further argue that brain glucose depletion underestimates the brain’s primacy over the rest of the body when it comes to energy and note (correctly) that during times of stress the amount of glucose supplied to the brain increases. This fact, they argue, makes the glucose model less likely, because if the body is able to supply glucose to the brain during times of stress, then it should be able to supply glucose to the brain during self-control exertion and hence avoid glucose depletion. It is true that glucose is released into the bloodstream at times of stress; indeed, much of the work on glucose and cognition builds on early studies that identified this glucose rise as a mechanism by which stress and the stress hormone epinephrine might exert nootropic effects on memory consolidation (e.g., see McGaugh et al., 1975; Gold et al., 1986; Hall and Gold, 1986; Gold and Stone, 1988; Talley et al., 2002). However, this effect has a time course on the order of minutes rather than the second or sub-second timeframe of acute increases in cognitive metabolic demand, and many increases in cognitive load occur in the absence of significant stress. Direct measurements suggest that the impact of stress-induced glucose provision to the brain following cognitive challenge is to replenish and reverse exhaustion of brain glucose rather than to prevent it (McNay et al., 2000; Sandusky et al., 2013).

The critique that brain glucose depletion is evolutionarily implausible is in error. However, a more limited version of this argument against the glucose model is, at first glance, more difficult to summarily dismiss. One might imagine, at least in theory, that vulnerability of self-control to reductions in glucose supply would be uniquely maladaptive. However, one could raise the same issue regarding all processes that are known to be limited by glucose availability (e.g., attention, memory, and so on) and no critic attempts to suggest that self-control is categorically more important to maintain than these processes.

To summarize, while it may not be evolutionarily adaptive for neural firing to cause local depletions in brain glucose, this does not serve as evidence that the brain always has plentiful glucose to support continued neural firing in those areas. Arguments against the evolutionary viability of brain glucose depletion have two inherent problems: (a) they oversimplify the complex nature of brain glucose metabolism; and (b) concepts such as developmental constraint provide a clear framework for traits that are not adaptive but are a product of past adaptations.

## General Defense of Brain Glucose Supply as a Constraint on Cognitive Functioning

It is *prima facie* plausible to suggest that brain glucose supply might always be sufficient to meet increased demand, at least under normal physiological conditions, as was suggested by early attempts to model brain glucose supply (Sokoloff, 1977; Lund-Andersen, 1979). However, this hypothesis faces two major obstacles. First, it is difficult or impossible to reconcile with the extensive literature on enhancement of cognitive function by glucose administration (Korol and Gold, 1998; McNay et al., 2000; McNay and Gold, 2002; Gold, 2005, 2014). Second, animal model studies that include direct measurement of glucose concentration in the brain’s extracellular fluid have consistently found that indeed, at times of increased cognitive demand, extracellular glucose decreases specifically in brains regions in which activity is increased and does so in proportion to the difficulty of the task being performed. This has been replicated by many different groups and is true across a wide variety of brain regions, treatments, and cognitive tasks (Fellows et al., 1992; Vahabzadeh et al., 1995; McNay et al. 2000, 2001, 2006a,b, 2013; McNay and Gold, 2001; McNay and Sherwin, 2004a; Canal et al., 2005; De Bundel et al., 2009; Rex et al., 2009; Newman et al., 2011; Pearson-Leary and McNay, 2012; Sandusky et al., 2013).

Moreover, manipulations that affect local glucose supply directly modulate cognitive performance, and delivery of additional glucose both prevents glucose depletion and enhances cognitive performance (e.g., McNay et al., 2000). Even relatively simple processes such as visual processing, to return to the example process chosen by Kurzban et al. (2013), have been repeatedly shown to cause localized depletion of glucose from brain structures that process visual input: presentation of a novel visual stimulus depletes specifically visual cortex glucose (Béland-Millar and Messier, 2018) while increasing local metabolism in a fashion very similar to hippocampal processing of a memory task; subsequent presentation of that same object (presumably requiring less processing) causes no such depletion, consistent with a task-difficulty explanation for depletion of local glucose. These and many other studies show very clearly that the first claim of the glucose model is likely correct: brain glucose is locally drained by difficult cognitive tasks and this depletion limits task performance. This finding is also in line with extensive literature in human populations with impaired glucose supply to the brain (e.g., aging, Alzheimer’s disease, or hypoglycemia) where cognitive impairment is similarly seen in proportion to the glucose supply decrease, and impairment is attenuated or reversed by provision of exogenous glucose just as it is in animal models (Hall et al., 1989; Manning et al., 1992, 1998; Cryer, 1994; Korol and Gold, 1998; Gold, 2005; Jauch-Chara et al., 2007).

Local, temporally limited depletion of glucose within specific brain regions that is caused by increased cognitive demand, and limits performance on tasks mediated by those regions, is now well established as fact: this component of the glucose model should no longer be controversial, despite the critiques of this element of the model.

## Self-Control as a Special Case

The second component of the glucose model is the claim that cognitive and neural processes involved in self-control are unique in causing exceedingly high demands for brain glucose use (Gailliot et al., 2007). Beyond suggesting depletion of brain glucose as a mechanism for impairments in self-control, they assert that glucose-limited cognitive function is a specific, unique characteristic of self-control rather than being a general characteristic of cognitive and neural processes. More concisely, these processes are argued to be “quantitatively different from other processes in that they require more glucose” (p. 306). This claim asserts that when individuals engage in self-control, they utilize highly energy-demanding areas of the brain that hence become drained of glucose, resulting in decreased ability to exert subsequent self-control under conditions that would not impair performance on other cognitive tasks.

The assertion of uniqueness for processes mediating self-control compared to all other cognitive functions is problematic, and the evidence does not support such a hypothesis. Rather, the impact of glucose administration and/or depletion on self-control appears to be very similar to that on other cognitive processes, consistent with the basic premise that all work requires energy. Glucose administration has been repeatedly found to enhance performance on a variety of cognitive processes such as memory and attention (the role of glucose as a modulator of these cognitive processes is reviewed in Gold, 1995, 2014). Furthermore, we suspect that most self-control tasks involve multiple cognitive processes, such that differentiating the relative cost of self-control is likely impossible. Arguments for the glucose model (e.g., Gailliot and Baumeister, 2007) neglect to note that glucose administration enhances a wide variety of cognitive and neural functions. There is a large literature over the past three decades showing, in a range of human and animal populations, that administration of glucose (regardless of the method of administration) can also enhance memory, attention, sensory processing, emotional regulation, mental flexibility, and a large number of other cognitive functions (e.g., Gold, 1986; Holmes, 1987; Hall et al., 1989; Michaud et al., 1991; Smith et al., 1992; McNay and Gold, 1998; Fucetola et al., 1999; McNay et al., 2000; Strachan et al., 2000; Benedict et al., 2006; Gajre et al., 2008; Benton and Brock, 2010; Micha et al., 2011; Jahagirdar et al., 2012; Sandusky et al., 2013).

There is no evidence in the literature for self-control processes being in any way different or unique. Such a difference has been asserted as the core of the glucose model. For example, Gailliot (2008) proposes that “[self-control is] more metabolically expensive and requires larger amounts of glucose to function optimally than other cognitive capacities” (p. 245), but no supporting evidence of any kind has been offered. This second key element of the glucose model has thus far been hypothesized in the absence of either theoretical or experimental support. Indeed, some of the canonical demonstrations that provide the theoretical basis for the original hypothesis that self-control is limited by depletion of cognitive resources, assumed to be glucose, rely on a commonality of limited resources (i.e., glucose supply to the brain) between self-control processes and those involved in, for example, memory (e.g., controlling visual attention leads to decrements in performing a working memory task; Schmeichel, 2007). This point appears to have been missed in formulations of the glucose model. One might imagine, at least in theory, that self-control processes could be a class of neural processes whose vulnerability to reductions in glucose supply would be uniquely maladaptive. However, one could raise the same issue regarding all processes that are known to be limited by glucose availability (e.g., attention, memory, and so on) and no critic attempts to suggest that self-control is categorically more important to maintain than these processes. Unfortunately, because there are as yet no data on direct measurements of brain glucose during a self-control task, we can conclude only that a limitation on such processes by glucose supply is plausible; we cannot and do not conclude that it is a fact. Such data would be difficult to obtain: techniques possible in humans such as fMRI do not directly measure glucose levels or metabolism (although PET is perhaps the closest thing); conversely in animal models where such measurements are possible, self-control is difficult to induce, especially for prolonged periods.

## Conclusion

Integrating data from metabolic studies that have examined brain glucose supply and usage with results from experiments investigating self-control can provide insights into mechanisms controlling the exertion of self-control. In particular, a wide variety of findings from diverse fields and using multiple approaches support the hypothesis that cognitive processing is limited by brain glucose supply especially under conditions of high cognitive demand, a conclusion that was well established across cognitive domains several decades prior to the formulation of the glucose model. This has been confirmed using direct measurements of brain glucose during cognitive tasks.

However, to the extent that self-control is posited to be special in this regard, there is no support in the literature for this position, despite the fact that it has attracted less criticism. The fact that many other cognitive functions have been shown to exhibit exactly congruent susceptibility to glucose depletion and enhancement by glucose supply suggests that there is nothing different about the processes underlying self-control. The conclusion drawn from these findings is that the second, novel component of the glucose model – the idea that self-control processes are in some manner uniquely metabolically demanding – is unsupported and likely false.

Although there is strong evidence to suggest that mental processes, including self-control, are fueled by glucose there are still many questions about glucose metabolism and its relationship to psychological outcomes. For example, the literature of self-control makes it clear that even after concentrated efforts of self-control, people may not give into temptation if sufficiently motivated (e.g., Muraven and Slessareva, 2003). This suggests that people may not fail at self-control because they run out of glucose. Likewise, research has found that consuming beverages that contain fructose, which takes some time to be metabolized into glucose (Gailliot et al., 2007), or even merely gargling but not swallowing sweet liquids can lead to better self-control performance (Hagger and Chatzisarantis, 2013). Thus, glucose metabolism on its own probably cannot explain the vagaries of mental performance.

These complexities suggest that theories focusing on just psychological models of mental effort or just biological approaches such as the glucose model of self-control depletion are too simple. In conclusion, we hope that a more nuanced and better-informed approach to the role of glucose in mental processes may take hold in the psychological literature.

## Author Contributions

BA and EM conceived the hypothesis and underlying framework. BA, MM, and EM discussed, refined, and wrote the manuscript.

## Conflict of Interest Statement

The authors declare that the research was conducted in the absence of any commercial or financial relationships that could be construed as a potential conflict of interest.
